# Grasping Preparation Enhances Orientation Change Detection

**DOI:** 10.1371/journal.pone.0017675

**Published:** 2011-03-08

**Authors:** Tjerk P. Gutteling, J. Leon Kenemans, Sebastiaan F. W. Neggers

**Affiliations:** 1 Department of Psychiatry, Rudolf Magnus Institute of Neuroscience, University Medical Center Utrecht, Utrecht, The Netherlands; 2 Department of Experimental Psychology, Utrecht University, Utrecht, The Netherlands; University of California, Berkeley, United States of America

## Abstract

Preparing a goal directed movement often requires detailed analysis of our environment. When picking up an object, its orientation, size and relative distance are relevant parameters when preparing a successful grasp. It would therefore be beneficial if the motor system is able to influence early perception such that information processing needs for action control are met at the earliest possible stage. However, only a few studies reported (indirect) evidence for action-induced visual perception improvements. We therefore aimed to provide direct evidence for a feature-specific perceptual modulation during the planning phase of a grasping action. Human subjects were instructed to either grasp or point to a bar while simultaneously performing an orientation discrimination task. The bar could slightly change its orientation during grasping preparation. By analyzing discrimination response probabilities, we found increased perceptual sensitivity to orientation changes when subjects were instructed to grasp the bar, rather than point to it. As a control experiment, the same experiment was repeated using bar luminance changes, a feature that is not relevant for either grasping or pointing. Here, no differences in visual sensitivity between grasping and pointing were found. The present results constitute first direct evidence for increased perceptual sensitivity to a visual feature that is relevant for a certain skeletomotor act during the movement preparation phase. We speculate that such action-induced perception improvements are controlled by neuronal feedback mechanisms from cortical motor planning areas to early visual cortex, similar to what was recently established for spatial perception improvements shortly before eye movements.

## Introduction

In daily life, there is a constant interaction between what we do and what we see. Our actions are partly dictated by what we perceive, but the reverse also holds. When performing an action, our perception is focused towards those things in our visual experience that enable us to execute the action successfully. For instance, when picking up a book, its orientation, thickness and distance all determine grasping kinematics early during the movement. When the perceptual system would be ‘primed’ towards relevant features, such as the orientation or size of the book, the subsequent grasping action can be executed with increased accuracy and speed.

This effect of motor preparation on visual perception has been well studied for the oculomotor system. It is now well established that, shortly before the actual execution of an eye movement, spatial perception greatly improves at the eye movement target location [1]–[3]. Furthermore, recent evidence demonstrates that oculomotor areas in the (pre)motor cortex influence processing in the visual cortex during eye movement preparation [4]–[6]. This is a likely neuronal mechanism underlying the observed links between spatial attention and eye movements.

It would make sense that when preparing more complex actions with the skeletomotor system, such as grasping and manipulating objects, not only spatial perception but also the perception of object features relevant for the task at hand would be improved. However, where there is ample evidence for this phenomenon in the oculomotor system, few reports exist for the skeletomotor system. Among the scarce reports there is encouraging, but indirect, evidence from the analysis of eye movement scanpaths before grasping [7] and the influence of subconscious priming on grasping reaction times [8] that indeed object orientation is perceived in an enhanced manner during grasping preparation. Although these few findings support the influence of action preparation on perception in the skeletomotor system, the measures used are speeded motor responses or eye movement scanpaths that might reflect interactions within the motor control system itself. As such, it is difficult to tease apart the contributions of the motor acts on perception and vice versa.

We therefore aimed to provide a direct measure of visual performance during (skeleto)motor preparation. This was done by estimating the visual sensitivity (d′) to slight orientation changes occurring during grasping and pointing preparation (just before the movement started). Visual discrimination performance was measured from non-speeded key presses well after the movement ended. Orientation was chosen as discrimination feature for its relevance for grasping acts, but not pointing acts. Should the preparation of a grasping act enhance the perception of relevant features, then sensitivity to orientation differences should be higher when preparing a grasping act, rather than a pointing act. As a control, luminance was chosen as a discrimination feature that is not relevant for either a grasping or pointing act. No differences in visual sensitivity were thus expected.

## Materials and Methods

### Ethics

This study was approved by the Medical ethical committee of the University Medical Center Utrecht. All subjects signed an informed consent prior to participation.

### Experiment I: Orientation

#### Participants

Sixteen subjects (11 women; mean age 25.9 SD4.4) with normal or corrected-to-normal vision participated in the first experiment. All were right handed, as checked by the Edinburgh handedness inventory (mean 85 SD22) [9].

#### Apparatus

Subjects sat in a dimly lit room in front of a IIyama 17″ (320×240 mm) monitor, with a resolution of 1024×768 pixels and a refresh rate of 100 Hz. They were seated in a frame with head- and chinrest. Viewing distance was adjusted to enable comfortable pointing and grasping movements. Visual angle of the stimuli was kept constant by compensating the size of the stimuli relative to the viewing distance.

To ensure that grasping and pointing actions were executed correctly, motion tracking of the right hand (grasping/pointing hand) was performed using a ‘driveBay’ magnetic motion tracker (Ascension technology, Burlington, USA). Subjects wore a flexible, unrestrictive glove that was fitted with four motion sensors located at the tip of the thumb, tip of the index finger, back of the hand and at the wrist. Movement data were recorded from all sensors at 240 Hz.

#### Task

**Figure 1:**
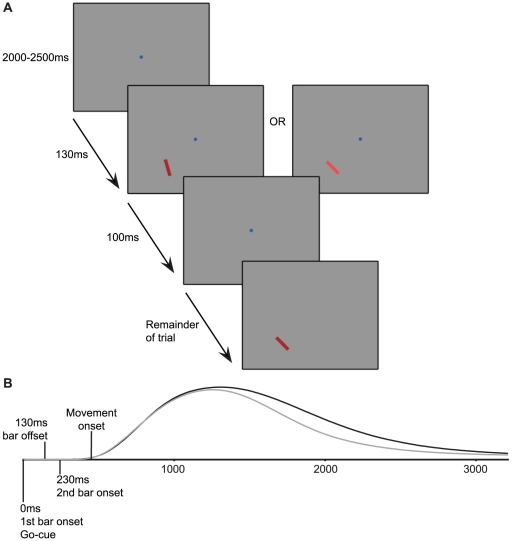
Experimental paradigm. (A) Stimulus display used in experiment 1 (orientation) and 2 (luminance). A fixation spot was followed by the appearance of a bar that signaled the go-cue for the action to be executed (by instruction) and which could be either rotated slightly (left, experiment 1) or differ in luminance (right, experiment 2) from the subsequent second bar. A brief fixation period (100 ms) was present between the first and second bar presentation. Subjects responded by key-press after execution of the action. (B) Timeline representation of the paradigm. The top plot represents the grand mean average movement (distance to origin) for either grasping (black) or pointing (gray).

Subjects were instructed to perform an orientation discrimination task, see Figure 1. Every trial started with a fixation spot (0.8° visual angle), after which a red rectangular bar appeared (4°×0.8° visual angle) in either of four locations, equidistant (8.5°) to the fixation spot. This bar stayed on screen for 130 ms. After a brief disappearance (100 ms) the bar reappeared in the same location, either slightly rotated or having the same angle. The 100 ms blank interval was added to ensure that the discrimination was made based on the difference in orientation and not as a consequence of the sudden transition between orientations, which induced a motion-like rotation which was much easier to detect. Subjects indicated by pressing a key with their left (non-dominant) hand whether they observed a difference in orientation between the first and second presentation of the bar. The bar stayed on screen until a response was given. Fixation was required until the bar reappeared (see above), the fixation spot disappeared at that moment. Simultaneously, subjects were required to perform either a grasp or point action to the appearing bar, depending on the instruction at the start of the block. The go-cue for this action was the first appearance of the bar. Subjects were specifically instructed to initiate the action as soon as the first bar appeared. This realizes a situation where the to-be discriminated orientation change occurs during grasping preparation, as the orientation change occurs 230 ms after the grasping/pointing go-cue, which is well before the grasping/pointing movement onset (pointing/grasping movements have latencies of around 400 ms [10], [11]. Grasping was performed by applying a precision grip in the length direction of the bar, i.e. to place index finger and thumb at the opposing short sides of the bar. The pointing action implied pointing to the center of the bar with the index finger.

The difference in orientation between the first and second bar could be either ‘none’, ‘small’ (2° rotation), ‘medium’ (4°) or ‘large’ (6°). The second bar was always oriented at either 45° or −45° (and hence the first bar at +/− 39, 41, 43, 45, 47, 49 or 51°). These differences occurred in both a clockwise and counter-clockwise direction. The second bar was always at the same orientation to avoid detection of the change after the movement preparation phase, as the second bar stayed on screen for the remainder of the trial. The magnitudes of change were small, as these were proven to evoke a stronger effect of action preparation in the pilot phase of the study. Movement onset time (>0.15 m/s) was monitored to check whether no movement was made before the second bar appeared, to ensure that the discrimination was made in the action preparation phase. In case this was violated, the trial was discarded.

Subjects were trained to reach adequate detection performance levels and were grasping and reaching properly before starting the actual experiment. On average, subjects completed 2–3 training blocks before starting the actual experiment. After training, subjects performed 4 blocks, each consisting of 64 trials. Grasping and pointing blocks alternated and were counterbalanced across subjects.

Stimuli were presented using custom software (‘Trackmagic’, written in C++) that was able to interface with the movement tracker for synchronized data acquisition. Care was taken to ensure accurate timing of stimulus presentation by synchronizing to the screen refresh rate of the display monitor.

#### Analysis

All computational analyses were done using customized Matlab scripts (The Mathworks, Natick, USA). Sensitivity (d′) for each ‘magnitude of change’ – ‘action type’ pair was estimated by subtracting z-transformed hit rates and block false alarm rate (d′ = Z(HR)−Z(FA), where HR = hit rate FA = false alarm rate and Z() is the z-transformation). Sensitivity (d′) represents how well one can detect a signal from noise, and thus is an estimation of the sensitivity to detect a certain stimulus, in this case an orientation change. This way, a measure of performance is obtained that is free of any response bias. For clarity, an indication of the response bias, in the form of log β, was also calculated (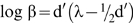, where d′ is the sensitivity and λ the response criterion -Z(FA)) [12].

**Figure 2:**
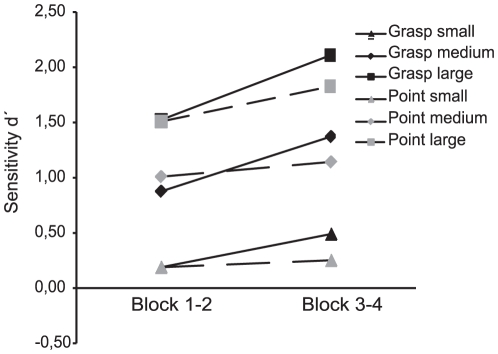
Effects of training. Separate analyses were performed on the first (block 1–2) and second half (block 3–4) of the first (orientation change) experiment. Differences in sensitivity due grasping or pointing preparation become apparent only in the second half of the orientation experiment (1).

The d′ values per condition were further analyzed in a repeated measures analysis of variance (ANOVA) with the factors ACTION (grasping/pointing) and CHANGE_MAGNITUDE (small/medium/large). The action counterbalancing order (grasping or pointing in the first block) was added as a covariate. A preceding pilot study showed that participants continued to show practice effects, even after training. These effects of training differed between grasping and pointing (see Figure 2). We therefore treated the first 2 blocks as further training. The results section therefore describes the results of the remaining 2 blocks, as this is considered as representative data without practice effects.

As all actions were performed only with the right hand, but stimuli appeared in both left and right visual field, there might be an effect of hemifield. To this end, in a separate analysis, the data were divided by visual field in which the bar appeared and collapsed over the magnitude of orientation change (to retain sufficient trials to give a reliable sensitivity estimate).

Additional parameters were extracted from the acquired movement data, including movement onset and duration, grasp angle and aperture. Movement onset threshold was set at 0.15 m/s. Trials with a movement onset before second bar appearance or two standard deviations beyond the subject mean movement onset were excluded from behavioral analysis, as it is unlikely that the discriminations in this case were made during action preparation.

The grasping angle was defined as the angle between the line defined by the DriveBay probes attached to the index finger and thumb, and an imaginary line along the vertical edge of the screen. This angle was extracted for every sample during every trial and sorted by target angle. For statistical testing purposes, angle timeseries were normalized to movement duration and divided in 25 equal time windows. A bonferroni adjusted significance threshold of p<0.002 was used.

All statistical analyses on the aforementioned parameters were performed using SPSS (15.0, SPSS inc., Chicago).

### Experiment II: luminance

Experiment 2 was identical to experiment 1, except as described below.

#### Participants

Sixteen subjects (13 women; mean age 25.6 SD3.5) participated in the second experiment, 12 of which had participated in experiment 1. All were right handed, as checked by the Edinburgh handedness inventory (mean 86 SD22) [9]. Subjects signed an informed consent prior to participation.

#### Task

The task was identical to experiment I, except the discrimination feature was luminance instead of orientation. Again, four levels of luminance changes were used: ‘none’, ‘small’ (±2.7%), ‘medium’ (±4.7%) and ‘large’ (±6.6%). These levels were chosen to match the difficulty of the orientation discrimination task, based on hit rates during grasping and pointing trials in the pilot phase of the experiment. Luminance levels of the first bar were either incremented or decremented, where the second bar always had the same luminance level.

## Results

### Orientation sensitivity

**Figure 3:**
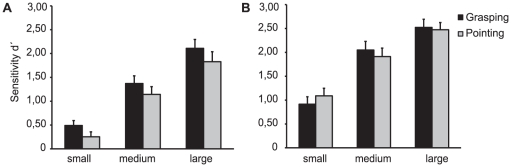
Main findings. (A) In the orientation change discrimination experiment (1), performance is increased when a grasping action is prepared. This effect occurs for all magnitudes of change tested. (B) No such consistent change in performance was found when luminance was used instead of orientation as a feature to-be discriminated.

We found that sensitivity to orientation changes increased when grasping, rather than pointing, see Figure 3 and Table 1. An analysis of (co)variance (ANCOVA) was conducted with factors ACTION (grasping/pointing), CHANGE_MAGNITUDE (small/medium/large change) and covariate ‘order’ (grasping or pointing first). This yielded a significant main effect of ACTION (F_(1,14)_ = 6.56, p = 0.023, partial η^2^ = 0.32), indicating that the visual sensitivity significantly differed, depending on the instruction to grasp or point. The mean overall sensitivity for grasping (1.32 SD 0.60; Hit rate 59.9% SD 16.1; false alarm rate 16.4% SD 14.4, bias log β 0.08 SD 0.53) was higher than the sensitivity for pointing (1.07 SD 0.63; Hit rate 57.9% SD 20.1; false alarm rate 22.6% SD 15.4, bias log β 0.15 SD 0.62).

**Table 1:** Behavioral performance for all conditions of experiment 1 (orientation).

Action/Change	Hits (%)	FA rate	d′	log β
*Grasping*		16.4		0.08
Small	32.1		0.49	(0.15)
Medium	62.9		1.37	(0.31)
Large	84.8		2.11	(−0.22)
*Pointing*		22.6		0.15
Small	31		0.25	(0.3)
Medium	60.3		1.14	(0.18)
Large	82.5		1.83	(−0.04)

‘Hits’: Percentage of correct detections. ‘FA rate’: Percentage of false alarms (indications of change when no change was present). ‘d′’: Measure of perceptual sensitivity. Log β: Measure of response bias towards either a change or no-change response.

Also, a significant main effect of CHANGE_MAGNITUDE (F_(2,28)_ = 24,82, p<0.001, partial η^2^ = 0.79) was found, showing that subjects sensitivity depended on the magnitude of the orientation change, as expected. No interactions between factors reached significance levels (ACTION×CHANGE_MAGNITUDE; F_(2,28)_ = 1.10, p = 0.51, partial η^2^ = 0.046).

**Figure 4:**
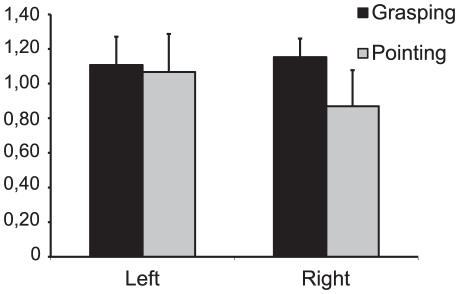
Visual hemifield differences in grasping and pointing performance. Differences in sensitivity between grasping and pointing are prominent when the stimulus is shown in the right visual field, but not when the stimulus appears in the left visual field.

To test for possible effects of visual field in which the bar appeared, a separate ANCOVA with factors HEMIFIELD (left/right), ACTION (grasp/point) and covariate ‘order’ was performed. This yielded a main effect of ACTION (F_(1,14)_ = 5.73, p = 0.031, partial η^2^ = 0.29) and a significant ACTION*HEMIFIELD interaction (F_(1,14)_ = 5.10, p = 0.040, partial η^2^ = 0.27), see Figure 4. This indicated that the effect depends on the visual field where the discrimination is made and the action performed. The sensitivity values in the left hemisphere show very little difference between actions (grasping: 1.11, pointing: 1.07), whereas the sensitivity values in the right hemisphere do (grasping: 1.15, pointing: 0.87). The increase in performance due to grasping preparation thus only seems to occur for stimuli in the right visual field, or the ipsilateral hemifield with respect to the performing hand.

Data rejection due to premature or late movement onset (beyond two standard deviations of the subject mean) was 3.0% (SD 1.5) on average. Rejection rates did not differ between actions (paired samples t-test, t(15) = 0.11, p = 0.91). Mean button press response time in the grasping condition was 1127 ms (SD 597) and 1096 ms (SD 568) in the pointing condition.

### Luminance sensitivity

As a control, the same experiment was repeated using luminance as the discrimination feature instead of orientation, see Figure 3 and Table 2. The same ANCOVA was performed as in the orientation experiment: ACTION (grasping/pointing), CHANGE_MAGNITUDE (small/medium/large) and covariate ‘order’ (grasping or pointing first). This yielded only a significant main effect of CHANGE_MAGNITUDE (F_(2,28)_ = 37.75, p<0.001, partial η^2^ = 0.85). No significant effect was found for ACTION (F_(1,14)_ = 0.40, p = 0.54, partial η^2^ = 0.027), or any interaction between factors (ACTION×CHANGE_MAGNITUDE; F_(2,28)_ = 0.44, p = 0.65, partial η^2^ = 0.035). Thus, no difference in visual sensitivity was found between grasping (mean d′ = 1.83 SD 0.67; hit rate 64.4% SD20.5; false alarm rate 7.3% SD8.3, bias log β 0.46 SD 0.62) and pointing (mean d′ = 1.82 SD 0.65; hit rate 65.2% SD19.7; false alarm rate 8.2% SD8.0, bias log β 0.53 SD 0.46) when using a feature that is not relevant for the action in preparation.

**Table 2:** Behavioral performance for all conditions of experiment 2 (luminance).

Action/Change	Hits (%)	FA rate	d′	log β
*Grasping*		7.3		0.46
Small	33.0		0.91	(0.72)
Medium	73.7		2.05	(0.60)
Large	86.5		2.52	(0.05)
*Pointing*		8.2		0.53
Small	38.1		1.09	(0.73)
Medium	70.1		1.91	(0.59)
Large	87.3		2.47	(0.27)

‘Hits’: Percentage of correct detections. ‘FA rate’: Percentage of false alarms (indications of change when no change was present). ‘d′’: Measure of perceptual sensitivity. Log β: Measure of response bias towards either a change or no-change response.

Again, an ANCOVA with factors HEMIFIELD, ACTION and covariate ‘order’ was performed. No significant main effects or interactions were found.

Data rejection due to premature or late movement onset (beyond two standard deviations of the subject mean) was 3.3% (SD 1.6) on average. Rejection rates did not differ between actions (paired samples t-test, t(15) = 0.0, p = 1.0). Mean button press response time in the grasping condition was 1100 ms (SD 692) and 968 ms (SD 569) in the pointing condition.

### Movement parameters

**Figure 5:**
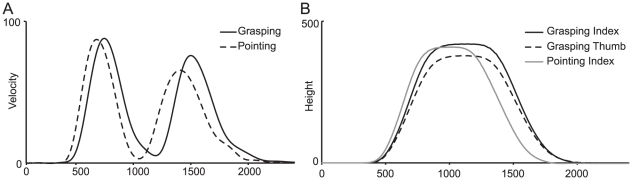
Kinematic data example. Exemplar data from grasping and pointing from a single subject, for a single bar position. (A) Velocity profile is taken from the wrist position. The first peak in velocity reflects the initial transport to the screen, whereas the second peak is caused by the retraction from the screen after the grasping/pointing action to the rest position. (B) Height profile is extracted from the thumb and index positions. Here, maximum height is reached when the subjects points to/grasps the bar on screen. Differences in thumb-index height in the grasping condition reflect the grasping aperture.

See Table 3 for an overview of the extracted movement parameters and Figure 5 for example kinematic data. No significant difference in movement onset was found between grasping and pointing (paired samples t-test, t = −1.16, p = 0.27) for the orientation experiment or for the luminance experiment (paired samples t-test, t = −0.55, p = 0.59). However, movement duration was significantly shorter for pointing (565 ms) than for grasping (610 ms) (paired samples t-test, t = 2.27, p = 0.039). This was also the case in the luminance experiment (paired samples t-test, t = 4.19, p = 0.001) for pointing (525 ms) and grasping (566 ms) durations.

**Table 3:** Mean movement parameters extracted from the movement tracker.

Action/Parameter (ms)	Movement onset	Movement duration	Angle preshaping
*Orientation*			
Grasping	550	610	>176
Pointing	576	565	NA
*Luminance*			
Grasping	517	566	>113
Pointing	513	525	NA

Movement onset times (ms) is the time between the go-cue (onset of the first bar) and the actual initiation of movement. Duration of movement (ms) is defined as the time between movement onset and movement offset (when the object on screen is grasped or pointed at). Angle preshaping time (ms) is the time point where a significant difference is observed in thumb-index angle between 45 and −45 degree target bars.

**Figure 6:**
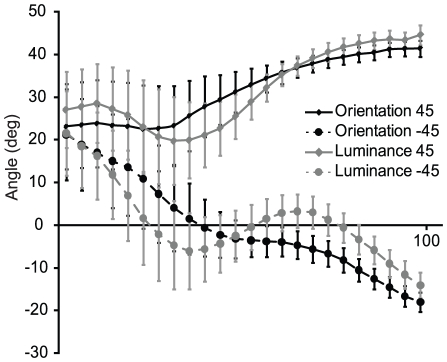
Grasping angle preshaping. Mean orientation of the thumb-index vector, as a function of target bar orientation (45 or −45 deg) and experiment (orientation/luminance) in the grasping condition. The horizontal axis represents the percent movement completed (0–100%), where 0% is movement onset and 100% is the point where the bar on screen is grasped. Error bars represent the standard error (SE).

To test for proper angle pre-shaping of the hand during grasping (that is, the alignment of the orientation of the hand with respect to the target in-flight), angle timeseries were separated for target angles of 45 and −45 degrees. These timeseries were divided in 25 time windows (where time windows 1 is movement onset and time window 25 is movement offset) and tested for significant deviation. This yields the time point in which the angle of the target bar influences the grasping action. The preshaping timecourse was averaged over all bar positions. For the orientation experiment, the difference between target angles reached significance from time window 7 (of 25) onwards (t = 4.97, p<0.001). This means pre-shaping of the hand was differentiated to target orientation from 28–32% of the grasping movement duration and onwards, which corresponds to 171–196 ms after movement onset, as the mean movement duration is 610 ms. In the luminance experiment it was slightly earlier, in time window 5 (t(15) = 3.92, p = 0.001; 20–24% of the grasping duration, 566 ms, or 113–136 ms after movement onset). See also Figure 6 for the time course of grasping preshaping to both bar orientations.

As the change in orientation may have influenced grasping angle preshaping, a similar analysis was performed on the angle timelines (divided over 25 time bins). Here, angle time courses were separated by orientation change condition (small, medium, large or none) and target angle (45 or −45 degrees) and tested for significant deviation as a function of magnitude of orientation change. In a CHANGE_MAGNITUDE(4)×TIME_BIN(25) ANOVA, no significant effect of CHANGE_MAGNITUDE was found for either the 45deg target orientation (F_(3,45)_ = 0.88, p = 0.46, partial η^2^ = 0.055) or the −45deg target orientation (F_(3,45)_ = 2.15, p = 0.11, partial η^2^ = 0.13), indicating that we found no influence of orientation change on grasping preshaping angle. Similarly, angle preshaping time courses were analyzed as a function of the given response (change/no change) instead of change magnitude. Again, no effect of the given response (45deg: F_(1,15)_ = 1.31, p = 0.27, partial η^2^ = 0.08; −45deg: F_(1,15)_ = 0.11, p = 0.74, partial η^2^ = 0.008) was found in the data.

## Discussion

In the current study, we found direct evidence for a perceptual enhancement of a specific, relevant feature when preparing a motor act. Visual sensitivity to object orientation change was increased when subjects prepared a grasping action (for which orientation is a relevant parameter) relative to preparing a pointing action (for which orientation is irrelevant). However, no differences in sensitivity were found between grasping and pointing preparation when a luminance change of the target object had to be discriminated, a feature that is irrelevant for both actions. Luminance is not an object feature that must be incorporated in a grasping action, unlike orientation. It is, however, an object feature that is similar to orientation for all other aspects of the task, thus controlling for non-specific effects. The critical difference between orientation and luminance is its relevance for the upcoming action. Take together with the existing literature, this strongly supports a specific action-relevant modulation of perception during action preparation (for encouraging results on grasping preparation and ‘size’ as the relevant feature, see [13], [14]). A direct measure of visual sensitivity was obtained by using non-speeded key-press responses occurring well after the grasping or pointing action, where the key-press reactions indicated a discrimination of visual changes that happened during action preparation. This way, we ensured there was no interference between two different active motor systems (key-presses and grasping/pointing) causing the observed influence of action on perceptual discrimination. This is supported by the finding that there was no influence of the magnitude of object orientation change on the preshaping angle of the hand during grasping and also, no difference in the grasping angle preshaping time course between ‘change’ and no-change' responses.

Analysis of the grasping and pointing movements revealed no differences in movement onset. This implies that the time course of the planning phase was similar, and that the discrimination was made during the same phase of action planning. An influence of target bar orientation (+ or − 45 degrees) on the grasping preshaping angle was found within the first third of the grasping movement, implying that the orientation of the object was an important factor in preparing the grasp.

Interestingly, the effect of action preparation on perception differed between visual hemifields. The effect of enhanced grasping performance was only present when the discrimination target was presented in the right visual field. This may be linked to the hand that was used to perform the action, which, in the current paradigm, was the right hand in combination with central fixation. Neuronal processes in motor related brain areas may only induce changes in perceptual areas within the same cerebral hemisphere, which might explain this effect (see the discussion of feedback based neuronal mechanisms below). One must note though, that there seems to be a non-specific overall increase in visual performance for both grasping and pointing movements in the left visual field. The improvement in discrimination performance for grasping movements we report here is always expressed relative to discrimination performance for pointing movements. This hemifield difference may be attributed to a general difference in performance between visual fields. It has been shown that different parameters of a visual stimulus (i.e. stimulus eccentricity, spatial frequency, perceptual demand) have differential effects on processing efficiency of the left and right hemisphere (for review, see [15]). In the current paradigm, the right hemisphere in right handed individuals (processing information from the left visual field) may be better suited to make the type of spatial discriminations required in the current task.

As mentioned in the method section and in Figure 2, training effects were still present after initial training and they seemed to differentiate between actions. To accommodate this, the first two blocks of the experiment were discarded and a ‘starting-action’ covariate (whether the subject started with either a grasping or a pointing block) was added to the analysis. It is interesting to note that the effects of action specific perceptual enhancement improved with training. It is likely that, especially for grasping, the artificiality of the current setup may have counteracted or occluded any performance gain at first. With practice, the actions became more automatic, as they are in daily life.

In general, discrimination performance during the luminance change experiment was slightly higher than during the orientation change experiment, despite efforts to match the difficulty. This may have impaired comparability between experiments. However, the orientation change experiment shows the effect of action preparation for all change magnitudes and thus seems independent of difficulty. Furthermore, the effect of action preparation does not show for the smaller change magnitudes in the luminance experiment, and therefore it is unlikely that the slight mismatch between orientation and luminance change discriminability explains why the effects of action-modulated perception were only found in the orientation change experiment.

Our current results agree well with existing literature. It fits with the idea that selection of visual processing (selective visual attention) is based on the intended action to be executed, that is, as a selection-for-action mechanism (e.g. [16]). This selection of action relevant information and the planning of this action may be implemented in a common mechanism (e.g. [17], [18]). This idea is closely related to the influential pre-motor theory of attention [19], stating that the preparation of a motor act is essentially identical to the attentional preparation that facilitates the action. Originally this was formulated for the oculomotor system, explaining covert shifts of attention as unexecuted eye movements. Later the theory was expanded to incorporate skeletomotor acts as well (e.g. [8]). In the latter study by Craighero et al., subjects had to execute a grasping action to a (real) bar object, triggered by a go-cue (a bar of matching or non-matching orientation) on a computer screen. When the bar on screen (go-cue) and the bar to-be-grasped had a matching orientation, movement onset times were reduced compared to incongruent orientations. The authors attributed this effect to enhanced visual processing of the go-cue due to the preparation of the grasping action. Although the latter explanation is likely, the results by Craighero et al. entail an enhanced orientation perception only for matching orientations. That is, the preparation of a grasping action facilitates the visual processing of objects with an orientation matching the prepared grasping action. This might be due to the specificity of the prepared grasping action, which was known before the appearance of the go-cue on screen in the study by Craighero et al. Therefore, this effect could also be related to a feed-forward process that perceives cues congruent to the instructed action in an enhanced way, rather than movement preparation effects that improve orientation perception.

In a similar fashion, Symes and colleagues [14] showed reduced reaction times in a change blindness paradigm if the changing object was congruent, rather than incongruent with a planned grasp (precision/power grasp and small/large objects).

The effect of action-modulated perception has also been shown to facilitate visual search for grasping-relevant features such as bar orientation. In a study by Bekkering and Neggers ([7], see also [20]), subjects had to grasp or point to an object of a certain orientation and color among other objects. The saccadic eye movements that naturally precede the grasping or pointing action were analyzed. Fewer eye movements were made to wrong orientations when subject had to grasp the object rather than point to it. Increased peripheral sensitivity to orientation, as was found in our study, can account for the performance improvement observed by Bekkering and Neggers. It is interesting to note that the visual search enhancement seemed to disappear with smaller set sizes. In the current study, three levels of difficulty were used, covering a wide range of performance. Here the effect did not disappear with decreasing difficulty, but remained consistent across difficulty levels. It may be that the current paradigm is more sensitive to performance differences, even when the task is relatively easy. Furthermore, the effect by Bekkering and Neggers can also be explained by interaction between two motor processes, grasping preparation and saccade scanpaths (patterns we are often not aware of). The present influence of grasping preparation on visual discrimination judgments, which we are fully aware of, cannot be explained this way.

One can speculate on the neuronal mechanism underlying action modulated perception. First, the current observed effect may be very similar to action-induced perceptual enhancements in the oculomotor system, where strong links have been found between spatial attention and eye movement preparations. Namely, Deubel and Schneider [1] showed that spatial attention is greatly increased at the target position of the upcoming eye movement. This has been interpreted as support for the influential pre-motor theory of attention [19]. Recently, the neural mechanisms underlying this effect have been studied in more detail. It is becoming clear that the effect is mediated by cortical feedback connections from the oculomotor areas (specifically the frontal eye fields) to occipital areas shortly before an eye movement [4]–[6], [21]. Such connections allow preparatory activation in motor control areas to modulate early visual processing in the occipital lobe. It may very well be so that this current form of ‘action-modulated perception’ is mediated by similar mechanisms in the skeletomotor domain. Cortical (pre)motor areas, specific for the action to be performed, might modulate visual processing through feedback connections to occipital areas. For instance, the anterior intraparietal area (AIP) would be an ideal candidate to fulfil such a function, as it is heavily implicated in the planning and execution of grasping actions [22], [23]. A recent EEG study [24] shows some initial evidence for such a mechanism. Here, grasping preparation elicited an enhanced occipital selection negativity that was absent in pointing preparation. This is indicative of an early modulation of visual processing specific to the preparation of a grasping action. Interestingly, when such feedback from AIP to the occipital lobe would occur within each cerebral hemisphere only, this would explain why we mainly find effects of grasping on perception in the right visual field. When assuming that left AIP is activated for grasping with the right hand, this induces changes in the left occipital lobe which in turn leads to action-modulated perception in the right (contralateral) visual field. Further studies are needed to unveil the exact neuronal mechanism driving the enhancement of action relevant features during action preparation.
